# Surgery

**Published:** 1878-08

**Authors:** 


### Surgery.

Artificial Emphysema as an Aid to Operation.—(*Archives de Méd. Navale*, No. 2, 1878.)

Dr. Roucière resorts to this measure to dissociate and isolate the different layers of superficial or deeper tissues, for the purpose of facilitating operations, such as discovery of vessels, enucleation of tumors, strangulated hernia, etc. In the hospital of Buenos Ayres it is thus performed: The skin is perforated with a trocar in the vicinity of the site of operation; to the canula is attached a suitable air pump apparatus. The tissues in the neighborhood are compressed, to avoid undue diffusion of the emphysema, and sufficient air driven in to distend the cellular tissue to the desired degree. The process is said to be free from bad results.

Compression of the Common Iliac.—Davy (*Br. Med. Jour.*, May, 18, 1878.

An account of a new method of compressing this vessel is related by Davy in a clinical lecture on amputation at the hip joint.

The rectum being empty, an enema of sweet oil was given. Then a smooth, straight, round lever of wood was introducep into the gut, the small end applied over the common iliac between the lumbar bodies and *psoas magnus* muscle, the projecting part of the lever being nearly parallel to the opposite thigh. The artery is easily compressed by elevating the projecting arm of the lever, the perinæal tissues acting as a lever. By this means the pulsations of the femoral may be caused to cease.

This is a less serious measure than compression of the abdominal aorta, the general circulation is far less disturbed, a simple straight lever suffices for apparatus, and no danger results to the rectum if the lever have been properly used. Mr. Davy also demonstrated how this method of compressing the iliacs, or even the aorta, might be utilized in many operations of pelvic surgery.

Amputation of Penis.—Stocks.—(*Br. Med. Jour.*, May 18, 1878.)

At the April meeting of the Manchester Medical Society, Mr. Stocks described a new method of operating, as follows: A narrow bladed knife is passed between the corpora cavernosa and the corpus spongiosum about an inch behind the diseased part and the former bodies divided directly upwards. The knife is then placed in the bottom of the wound and carried directly forward about four lines, and then diagonally forwards and downwards, forming an inferior flap. The urethra in this locality is then freed from the corpus spongiosum and slightly loosened from its attachments. The skin flap is turned upwards, a small button hole made in it through which the loosened urethra is drawn by forceps and so held until the flap is adjusted and secured. The cut end of the urethra is afterwards stitched to the edges of the button hole. Any redundancy of integument forms an admirable foreskin to protect the tender urethral orifice, and is easily drawn back so as not to form impediment to micturition.

Mr. S. exhibited a case in which this method had proved quite successful.

Nerve Section for Elephantiasis. — Morton. (*Phila. Med. Times*, Jan. 19, 1878.)

Dr. Morton, during December, '74, tied the femoral artery of a colored patient suffering from an extreme case of elephantiasis arabum. The patient was discharged in March much benefited.

He returned, however, Nov., '77, for further treatment, desiring an amputation, the leg measuring 21 inches in circumference. Having noticed the frequency with which nerve section is followed by atrophy of parts supplied by that nerve, Morton determined to attempt the production of artificial atrophy in this case. Accordingly the right sciatic was laid bare, and an inch and a half of its length exsected from the upper part of the thigh.

No untoward symptoms followed. There has been a steady diminution in size ever since, and the thick skin has desquammated, leaving soft pliable skin beneath, while there has been no tendency to ulceration.

This is, so far as known, the first conception and performance of this comparatively simple means of effecting relief in a most troublesome and loathsome malady.

New Method of Treating Varicocele.—Bradley. (*Br Med. Jour.*)

The author explains his process as follows: Pass a long and strong hare-lip pin between the veins and scrotal walls, bringing the point close beneath, but not through, the scrotum; then make the point retrace its course, but passing now behind the veins until it emerges near the puncture through which it entered. The varicocele may thus be effectually compressed, and the veins obliterated.

Abortive Treatment of Bubo.—Malplaquet. (*L'Union Médicale*, *No.* 53, 1878.

By means of vesicant fluid, the epidermis is removed the size of a franc over the seat of the swelling. Upon this denuded surface a piece of lint is laid, soaked in a saturated solution of bi-chloride of mercury, and over the whole is laid a flaxseed poultice, which is left on about twenty-four hours. At the end of this time a gray eschar is seen, and after the poultices have been applied two or three days, there only remains a slight depression with granulated surface which heals readily by simple dressings. The mercurial lotion gives sharp pain for a short time, but in twelve cases treated in this way the best of results have been obtained.

Osteo-myelitis during Growth. M. Lannelong. (*Bull. de l'Acad. de Méd. No.* 22).

1st. The affection described by authors under the names of acute necrosis, acute periostitis, phlegmonous, epiphysiary osteitis, etc., is in reality acute osteo-myelitis.

2d. The long bones are most exposed to it, but the short bones are also exposed.

3d. In the long bones the primitive seat is found in the line of union of the diaphyses with the epiphyses; the cartilage remains intact in 15 to 20 per cent. of the cases.

4th. One of the earliest consequences is a separation of the periosteum with subperiostic abscess.

5th. Parallel with the necrosis and thinning of the bone, a work of reparation takes place which ends in the formation of a new bone.

6th. Articular complications do not always exist; their appearance renders the prognosis of the affection more grave.

7th. When the diagnosis is established, trephining is the only method of which the opportunity and the indications are undeniable.

Drainage of the Bones in Necrosis and Osteo-Myelitis. Armand Deprés. (*L'Union Médicale, May* 23, 1878, p. 787.)

1st. In osteo-myelitis with spontaneous fracture of the long bones, whenever the articulations are intact, the limb should be preserved by the aid of an incision reaching to the bone, according to the precepts of Smith, Broca and Gosselin, by removing constrictions over the abscesses and by passing in a drain to the seat of fracture through the open abscesses around the bone.

2d. The drain should be left in place a year, so as to place the central necrosis of the bone in the condition of a superficial necrosis, at the bottom of a wound of the integuments with loss of substance.

3d. The drainage of bones affected with osteo-myelitis, like disarticulations practiced in the same conditions, as an operation which may be done while the patients have fever; but the gravity of the drainage, equaling that of an opening of an abscess, is less than that of disarticulation, and aside from other reasons should cause the drainage to be preferred.

Ergotine in Strangulated Hernia.—(*L'Union Médicale du Canada, June* 1878, p. 262.)

Two cases of strangulated hernia successfully treated with ergotine are reported in *Bordeaux Médicale* (author's name not given). In both cases other means had failed, and an operation was contemplated.

The skin over the tumor was first washed with an alkaline lotion to facilitate absorption, and then annointed with pure ergotine every two hours, at the same time tablespoonful doses of a solution containing ʒiss of ergotine to ℥iv of the vehicle, were given every hour. Four or five hours after beginning treatment the vomiting and pain lessened and the tumor diminished, and in twelve hours the hernia reduced itself. The author attempts no explanation of the mode of action of ergotine in these cases, but recommends further trial.

Application of Ligatures after Castration.—Novel method of performing phimosis.

In the surgical section of the Parisian Academy of Sciences, under the Presidency of M. Tarnier, the general subject of ligation after the removal of the testicle was discussed. Four of the six surgeons who took part in the discussion, gave their opinion decidedly in favor of the separate ligation of the vessels. The objections urged to the tying *en masse* were:

The danger of tetanus; tumefaction of the cord, and the greater time that elapses before the ligature becomes disengaged.

In the same section, Dr. Hue, of Rouen, read a paper on the performance of phimosis by an elastic ligature. He passes a needle, armed with an elastic thread, through the prepuce at the base of the glans penis on the dorsal surface; then ties it, comprising in the ligature that part of the prepuce in front of the perforation. In three or four days, says M. Hue, the section is complete. There is very little pain. He has seen the operation performed in all, eighteen times, and it is equally applicable to children or adults. In point of æsthetics, says M. Hue, the final result is more elegant.

